# Extracting more light for vertical emission: high power continuous wave operation of 1.3-μm quantum-dot photonic-crystal surface-emitting laser based on a flat band

**DOI:** 10.1038/s41377-019-0214-2

**Published:** 2019-11-22

**Authors:** Huan-Yu Lu, Si-Cong Tian, Cun-Zhu Tong, Li-Jie Wang, Jia-Min Rong, Chong-Yang Liu, Hong Wang, Shi-Li Shu, Li-Jun Wang

**Affiliations:** 1 State Key Laboratory of Luminescence and Applications, Changchun Institute of Optics, Fine Mechanics and Physics, Chinese Academy of Sciences, 130033 Changchun, China; 2 The University of Chinese Academy of Sciences, 100049 Beijing, China; 3 Bimberg Chinese-German Center for Green Photonics, Changchun Institute of Optics, Fine Mechanics and Physics, Chinese Academy of Sciences, 130033 Changchun, China; 4 National Key Laboratory for Electronic Measurement Technology, School of Instrument and Electronics, North University of China, 030051 Taiyuan, China; 5 Temasek Laboratories, Nanyang Technological University, 50 Nanyang Drive, 637553 Singapore, Singapore; 6 Nanyang Technological University, 50 Nanyang Drive, 637553 Singapore, Singapore

**Keywords:** Semiconductor lasers, Micro-optics

## Abstract

For long distance optical interconnects, 1.3-μm surface-emitting lasers are key devices. However, the low output power of several milliwatts limits their application. In this study, by introducing a two-dimensional photonic-crystal and using InAs quantum dots as active materials, a continuous-wave, 13.3-mW output power, 1.3-μm wavelength, room-temperature surface-emitting laser is achieved. In addition, such a device can be operated at high temperatures of up to 90 °C. The enhanced output power results from the flat band structure of the photonic crystal and an extra feedback mechanism. Surface emission is realized by photonic crystal diffraction and thus the distributed Bragg reflector is eliminated. The proposed device provides a means to overcome the limitations of low-power 1.3-μm surface-emitting lasers and increase the number of applications thereof.

## Introduction

Surface-emitting lasers (SELs) operating at 1.3-μm are key devices for optical interconnects^1,2^ spanning distances exceeding kilometers in length, and are crucial for low-cost high-capacity optical interconnects between data centers due to advantages including ease of testing and integration, low fabrication cost, and good beam quality with a circular beam. Several gain media emit at 1.3-μm. GaAs-based materials show more advantages than InP-based materials due to growth compatibility with AlGaAs/GaAs Bragg reflectors. Furthermore, compared with GaAs-based quantum wells (QWs)^3,4^, In(Ga)As/GaAs quantum dots (QDs)^5–11^ are more promising because of the 3D carrier confinement, demonstrated low-threshold current densities and high characteristic temperatures^12–14^. Hence, 1.3-μm QD SELs are ideal candidates for long-distance optical interconnects.

SELs can be realized with vertical-cavity surface-emitting lasers (VCSELs) grown on a microcavity of an integer number of half-wavelengths using distributed Bragg reflectors (DBRs)^5–9^. However, the output power of 1.3-μm QD VCSELs under continuous-wave (CW) operation at room-temperature (RT) is at most 2.2 mW^7^, because of the complex and critical doping, growth of the DBR, oxide-confined structure, and hence poor thermal resistance. Although high power VCSELs with large apertures can be obtained, the higher-order modes resulting from the larger cavity critically degrade the beam quality.

Photonic-crystal surface-emitting lasers (PCSELs), which are based on band-edge effects of the photonic crystal (PC), realize self-mode locking (SML) by diffraction^11,15–22^. The strong loss dispersion at the Brillouin zone center in PCs enables single-mode PCSELs with a large area^22^. Although, PCSELs can be viewed as a variation of grating coupled SELs^10,23^, the multidirectional Bragg diffraction in PCSELs can yield coupling mechanisms unattainable with grating-coupled SELs, leading to control of the lasing mode over a large 2D area. In addition, the polarization^17^, beam pattern^18^, and direction^19^ of PCSELs can also be controlled. In contrast to VCSELs, surface emission is realized by PC diffraction, thus, no DBR is needed, which is especially suitable for 1.3-μm SELs. However, the power of 1.3-μm QD PCSELs is still limited to 2 mW when operated in pulsed mode at RT^11^.

PCs form energy bands for photons, which is an important attribute for PCSELs. A perfect or partial flat band is a dispersionless band structure^24–26^. Such a feature can provide slow light with zero group velocity and a high density of states over a broad range of the Brillouin zone. Therefore, enhanced light–matter interactions can be realized in such a range where a flat band exists^27^. In a previous study, a flat band was only realized at the second-order Gamma point, where a flat band mode exists and can support lasing of PCSELs^28^.

Inspired by the above works, in this study, the flat band of a PC is engineered to realize a high power 1.3-μm InAs QD PCSEL. In addition, an extra lateral feedback mechanism is introduced to further improve the output power of the PCSEL. The CW output power of the PCSEL at RT is 13.3 mW, which is higher than the output reported for any other 1.3-μm InAs QD SEL. Furthermore, such lasers can be operated at high temperatures, meeting the demands of optical communication applications.

**Fig. 1:**
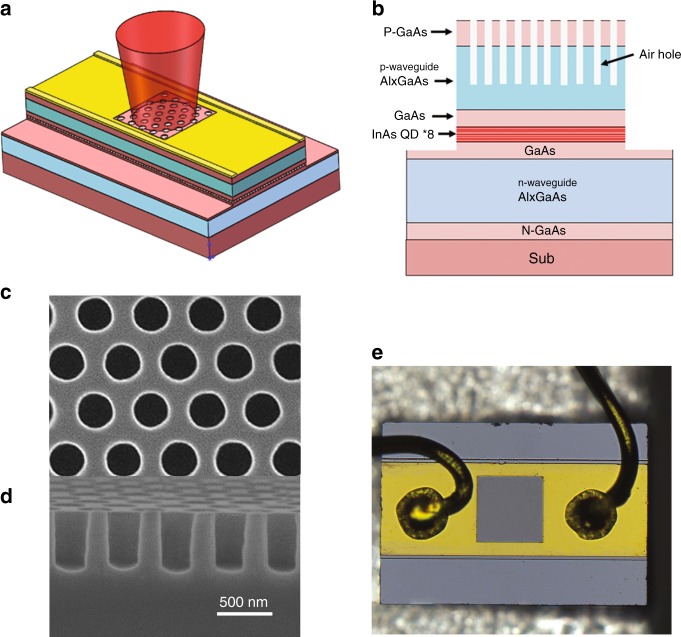
Schematic drawing and optical micrograph of the PCSEL and SEM images of a PC. **a** Schematic of the PCSEL. **b** Cross-sectional schematic of the PCSEL. **c** Top-view SEM image of the 2D PC. **d** Side-view SEM image of the 2D PC. **e** Top-view optical micrograph of the PCSEL.

A schematic of the structure of the PCSEL and its cross-section are shown in Fig. 1a, b, respectively. Light traveling in the slab is diffracted along the slab surface normal direction by the PC, forming a surface-emitting laser. Different from previous PCSELs, here we introduce a waveguide to form a Fabry–Pérot (FP) cavity, which can provide extra lateral feedback and a large area for current injection. The device width, length, and depth are 280, 800, and 2 μm, respectively.

The PC region is 200 × 200 μm^2^, consisting of a triangular lattice in which each lattice point corresponds to a hole with a circular cross-section. Top-view and side-view scanning electron microscopy (SEM) images of the PC are shown in Fig. 1c, d, respectively. The lattice constant of the PC structure *a* is 520 nm. The diameter and depth of the holes are 300 and 800 nm, respectively. The holes were fabricated above the active layer, which favors realization of a high output power^29^. An overview top-view optical micrograph of the PCSEL is shown in Fig. 1e.

**Fig. 2:**
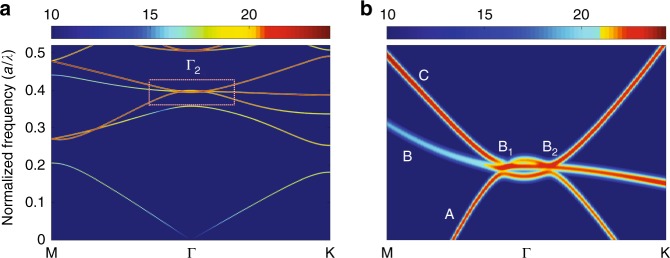
Calculated TE band structure of the 2D PC. **a** TE band structure of the 2D PC. **b** TE band structure of the 2D PC in the vicinity of the Γ_2_ point.

We calculate the transverse-electric (TE) band structure of the PC as shown in Fig. 2a, and then the band structure around the second-order Γ-point (Γ_2_) as shown in Fig. 2b. It is known that the mode with the highest *Q* value is favored for lasing. Thus the *Q* factor of each mode is calculated (Table S1 of the Supplementary Information), and it is found that mode B has the largest *Q* factor near the Γ_2_ Point. Therefore, mode B is more likely than the other modes to become the lasing mode. As shown in Fig. 2b mode B exhibits a flat band around Γ_2_ (between B1 and B2). In this flat band region, the group velocity of light becomes zero and a standing wave is formed. Such an unusual PC band structure is quite different from that of previous studies, in which the flat band only exists at Γ_2_^28^. As a result, strong interaction between light and matter occurs not only at Γ_2_, but also in the vicinity of the flat band, which is the main factor for realizing a high output power of the PCSEL.

**Fig. 3:**
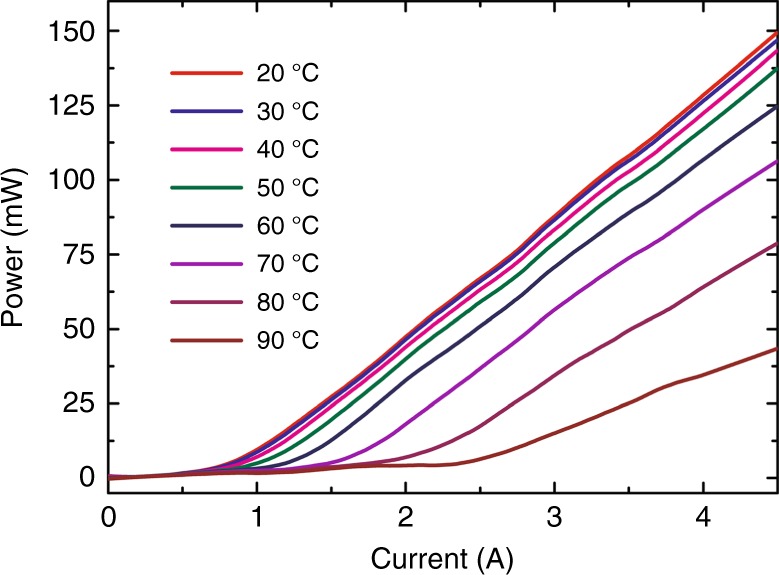
Laser characteristics of the PCSEL under pulsed operation. Light-current characteristics for temperatures from 20 to 90 °C for a drive current modulated at 5-kHz with a pulse duration of 2 μs.

We next discuss the lasing characteristics of our PCSEL under pulsed operation from 20 to 90 °C. Under these conditions, thermal effects can be suppressed. We show the light–current characteristics for varying temperatures in Fig. 3. The maximum output power of the PCSEL is 150 mW at a current of 4.5 A, at 20 °C (RT). Under these conditions, the threshold current *I*_th_ and slope efficiency are 0.86 A and 40.9 mW A^−1^, respectively. As the operating temperature is increased, the output power and the slope efficiency of the laser decrease, and the threshold of the laser increases. It should be noted that the change in the light-current curves in the temperature range of 60–90 °C is larger than that in the temperature range 20–50 °C.

**Fig. 4:**
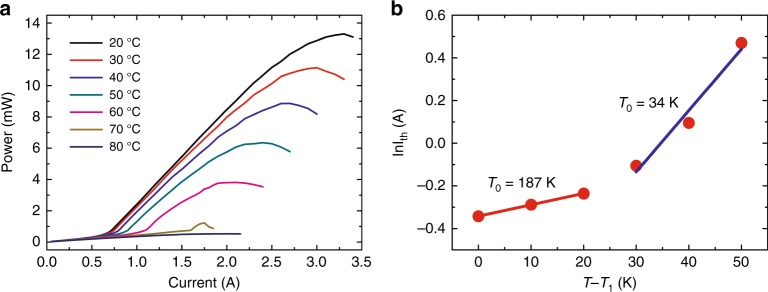
Laser characteristics of the PCSEL for CW operation. **a** Light-current characteristics for varying temperatures from 20 to 80 °C in steps of 10 °C. **b** The characteristic temperature as a function of the threshold current *I*_th_ of the PCSEL.

Next, the lasing characteristics of the PCSEL under CW operation are discussed. The measured output power of our PCSEL as a function of the injection current for various operating temperatures is shown in Fig. 4a. When the operating temperature is 20 °C (RT), the light-current curve exhibited thermal roll-over characteristics. The maximum output power of the device is 13.3 mW at 3.2 A, which is five times larger than that of previously reported InAs QD SELs^7,10,11^. The threshold current is 0.71 A, corresponding to a current density threshold of 48.2 mA cm^−2^ per layer of QDs. The slope efficiency of the laser is 5.8 mW A^−1^. With increasing operating temperature, the saturated output power and the slope efficiency decrease, and the threshold increases. However, when the temperature exceeds 70 °C, the laser stops working normally due to thermal effects. Furthermore, in Fig. 4b we show the impact of the operating temperature on the threshold current *I*_th_. From this curve one can calculate the characteristic temperature *T*_0_ of the PCSEL (Section 5 of the Supplementary Information), that is, 187 K in the range of 0–20 and 34 K in the range of 30–50 K.

**Fig. 5:**
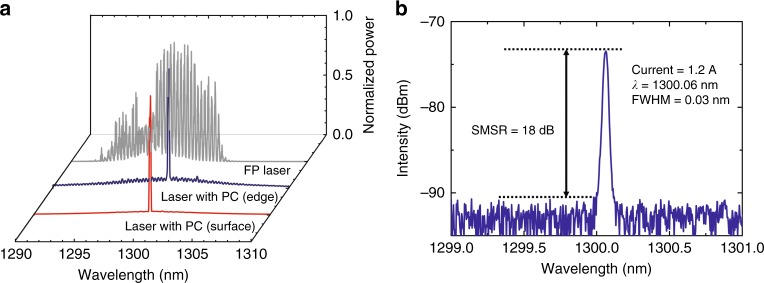
Laser spectrum of the PCSEL operating CW at RT. **a** The lasing spectrum of surface emission of the PCSEL (red line), edge emission of the PCSEL (blue line), and edge emission of the FP laser without a PC structure (gray line), respectively. **b** The spectrum of the surface-emitting PCSEL.

Last, we measure the spectrum of the PCSEL operating in CW mode at RT. As shown in Fig. 5a, the FP laser without a PC structure exhibits multimode output, while the PCSEL exhibits single mode output for both surface and edge emission. This phenomenon occurs because the PC can modulate the modes, suppressing the comb modes of the FP cavity and enhancing the PC mode around the Γ_2_ band edge. The spectrum of the surface emission of the PCSEL under a current of 1.2 A is shown in Fig. 5b. It can be seen that the PCSEL exhibits single-mode emission. The center wavelength and full-width at half-maximum (FWHM) of the spectrum are 1300.06 and 0.03 nm, respectively. In addition, the side mode suppression ratio (SMSR) is 18 dB. However, under a higher current, the PCSEL will exhibit multimode operation.

In conclusion, we have achieved a 1.3-μm high power InAs QD PCSEL. We achieved an output power of 150 mW for pulsed operation at RT. The device can be operated in pulsed mode at temperatures up to 90 °C. More importantly, we achieved an output power of 13.3 mW for CW operation at RT. This output power is five times larger than of that of previous InAs QD SELs. The device can be operated in CW mode at temperatures up to 60 °C. The higher output power of the PCSEL occurs because of the flat band of the PC slab in the Brillouin zone and the introduced waveguide. Our fabrication process is simplified, reducing costs, because the need for DBR growth, epitaxial regrowth, and wafer fusion bonding is eliminated. Our devices provide a route towards realizing high-power 1.3-μm SELs that can be widely used in optical communications and for optical interconnects in super computers and data centers.

## Methods

### Fabrication

Using molecular beam epitaxy (MBE), layers were grown on an n-type GaAs (001) substrate. The details of the structure of the resulting QD sample are provided in Section 1 of the Supplementary Information.

The PC structure was fabricated on the QD sample. After depositing a 300-nm SiO_2_ etching mask by plasma-enhanced chemical vapor deposition (PECVD), a 2D PC was fabricated via electron-beam lithography followed by two ICP dry etching steps.

After fabrication of the PC structure, the PCSEL was fabricated by the conventional edge-emitting-laser fabrication process. The samples with a PC structure were patterned into stripe lasers by photolithography and ICP dry etching. A 200-nm thick SiO_2_ electrical insulating layer was deposited by PECVD at 300 °C, and a contact window opening was fabricated within the PC area by photolithography and ICP dry etching. Standard p-side metallization was performed via a lift-off process, and finally, after the processes of substrate thinning and metallization of the n-side, a single device was obtained.

### Simulation

Simulations were performed by the finite-difference time-domain (FDTD) method to investigate the band structure of the PC slab. Here, only TE modes were focused on, because the gain of the TE mode is larger than the gain of the transverse magnetic (TM) mode. The band structure around Γ_2_ was investigated at a higher resolution, because in the vicinity of Γ_2_, the PC will supply distributed feedback and couple the light into the normal direction.

### Measurement

The light-current characteristics were measured by a thermal-type power meter, which was placed in front of the PC area normal to the surface of the PCSEL. The temperature was controlled by thermoelectric cooling (TEC). The width and delay of the pulse were 2 μs and 20 ms, respectively.

The spectrum was measured at a current of 1.2 A, and the laser beam was collected by a single-mode fiber coupled to an optical spectrum analyzer (Yokogawa AQ6370C).

## Supplementary information

Supplementary information
