# Supplementary material for: Extracting more light for vertical emission: high power continuous wave operation of 1.3-μm quantum-dot photonic-crystal surface-emitting laser based on a flat band
Source: Light Sci Appl. 2019 Nov 22;8:108. doi: 10.1038/s41377-019-0214-2 (PMC6874546; doi:10.1038/s41377-019-0214-2)
Supplement: Supplementary file 1 — Supplementary information [file 41377_2019_214_MOESM1_ESM.docx]

**Supplementary information**

**Extracting more light for vertical emission: high power continuous wave operation of 1.3-μm quantum-dot photonic-crystal surface-emitting laser based on a flat band**

Huan-Yu Lu^1, 2^, Si-Cong Tian^1, 3^, Li-Jie Wang^1^, Jia-Min Rong^4^, Chong-Yang Liu^5^, Hong Wang^6^, Shi-Li Shu^1^, Cun-Zhu Tong^1^, and Li-Jun Wang^1^

Correspondence: Cun-Zhu Tong ([tongcz@ciomp.ac.cn](mailto:tongcz@ciomp.ac.cn)) or Si-Cong Tian (tiansicong@ciomp.ac.cn)

^1^State Key Laboratory of Luminescence and Applications, Changchun Institute of Optics, Fine Mechanics and Physics, Chinese Academy of Sciences, Changchun 130033, China

^2^The University of Chinese Academy of Sciences, Beijing 100049, China
^3^Bimberg Chinese-German Center for Green Photonics, Changchun Institute of Optics, Fine Mechanics and Physics, Chinese Academy of Sciences, Changchun 130033, China

^4^National Key Laboratory for Electronic Measurement Technology, School of Instrument and Electronics, North University of China, Taiyuan 030051, China

^5^Temasek Laboratories, Nanyang Technological University, 50 Nanyang Drive, 637553, Singapore

^6^Nanyang Technological University, 50 Nanyang Drive, 637553, Singapore

**1. Structure of 1.3-μm InAs/GaAs quantum dot sample.**

The wafer investigated in this study was grown on an n-type GaAs (001) substrate by molecular beam epitaxy (MBE). The layer structure is shown schematically in Figure S1. A 300 nm n-doped GaAs buffer layer was grown on the GaAs substrate, followed by a 1440 nm n-doped bottom cladding layer of Al_x_Ga_1-x_As, and a 60 nm undoped GaAs separate confinement heterostructure (SCH) layer. Next an undoped active region consisting of eight layers of InAs QDs was grown. Another 20 nm undoped GaAs SCH layer, a 1400 nm p-doped top cladding layer of Al_x_Ga_1-x_As, and a 400 nm p-doped GaAs contact layer were grown on top of the active region.


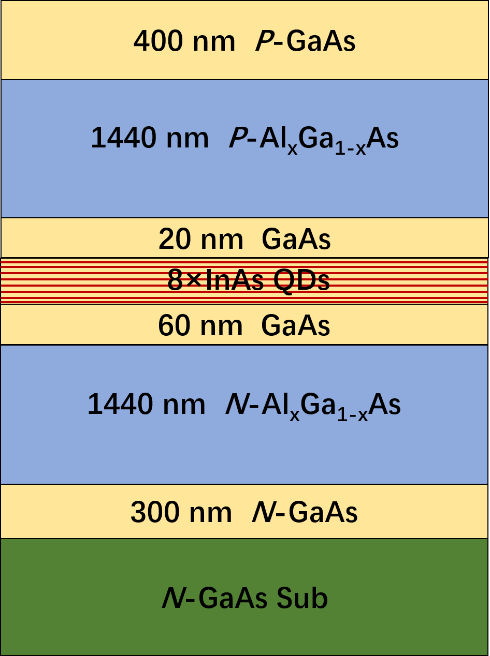


**Supplementary Figure S1. Schematic layer structure of the InAs/GaAs QD sample.** Al content x of the cladding layers, and typical dopant density of n-doped and p-doped layers can be varied.

**2. The vertical emission characteristics of photonic crystals**


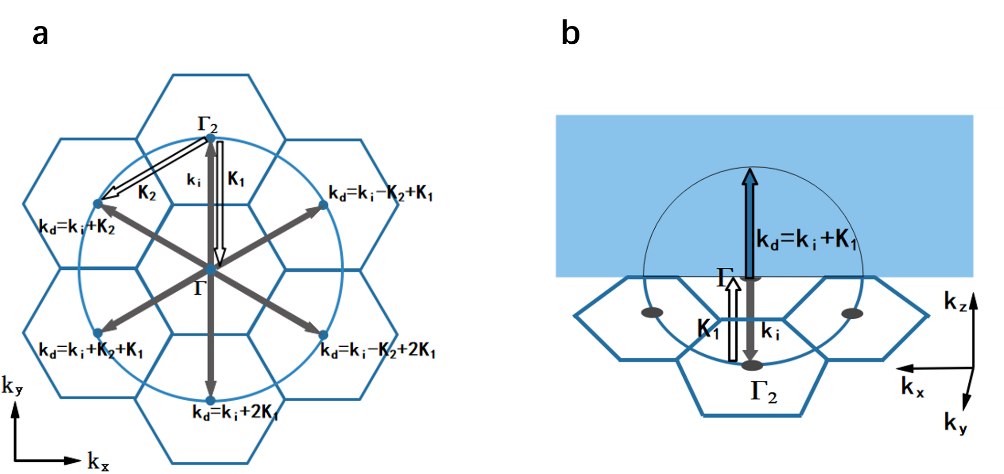


**Supplementary Figure S2. Wave-vector diagram and Bragg diffraction conditions at Γ2.** a, In-plane, and b, out of plane.

A diagram of the wave-vector and the conditions for Bragg diffraction are shown in Figure S2. $\vec{k}_{d}$ and $\vec{k}_{i}$ are the in-plane component wave vectors of the diffracted light and the incident light, respectively. $\vec{K}$**_1_** and $\vec{K}$**_2_** (outline arrows) are the Bragg grating vectors, and $\left| \vec{K} \right|=2п/a$ is the magnitude, where *a* is the lattice constant. For Γ2 points, the incident light is diffracted in five different Γ-X directions and one direction normal to the slab. The diffracted light, which is normal to the slab, results in a surface emitting mode. The relations between the diffracted light and the incident light under the Bragg diffraction condition can be seen in Figure S2, which meets the phase matching condition [1].

**3. The flat band of the photonic-crystal**

By using the finite-difference time-domain (FDTD) method, we calculate the Q factors of the three modes of the two-dimensional photonic crystal with 13 airholes in the Γ-M direction. The results are shown in Table S1. As can be seen the Q value of mode B is larger than those of modes A and C. Therefore, mode B is the most likely to result in lasing.

| Mode | A | B | C |
| --- | --- | --- | --- |
| Q factor | 166 | 197 | 184 |

**Supplementary Table S1. Q factors of the bands near 1.3 μm.**


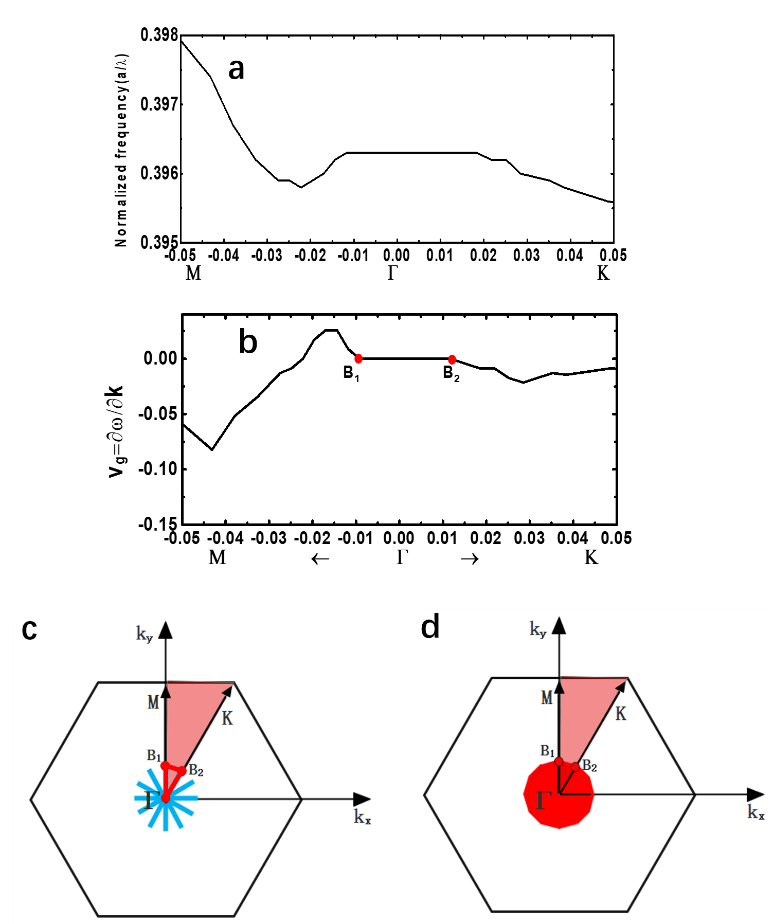


**Supplementary Figure S3.** **Band structure and group velocity of mode B, and the area of flat band.** a, The detailed band structure of the mode B. b, The group velocity of mode B near the point Γ. c, The area of the flat band in the irreducible Brillouin zone. d, The area of the flat band in the first Brillouin zone.

Surprisingly, by tuning the radius of the holes of the two-dimensional photonic crystal consisting of a triangle lattice of circular air holes, it is possible to realize a flat band of mode B (Figure S3a), where the group velocity of the field is zero (Figure S3b). In the irreducible Brillouin zone, light will be localized in the triangle (Γ-B1-B2) shown in Figure S3c. Therefore, in the first Brillouin zone, strong interaction between light and matter will occur in a larger area (red dodecagon in Figure S3d), which is responsible for the larger output power of the PCSEL.

**4. The effect of the Fabry-Pérot (FP) cavity**

The high reflectivity of the side boundary of the PCSEL is beneficial to the modal frequencies and corresponding threshold gains [2]. However, etched boundaries usually do not provide high reflectivity (Figure S4a), whereas the cleaved sides of the FP cavity provide high reflectivity (Figure S4b) [3,4]. In addition, more gain materials are involved and contribute to the increased output power.


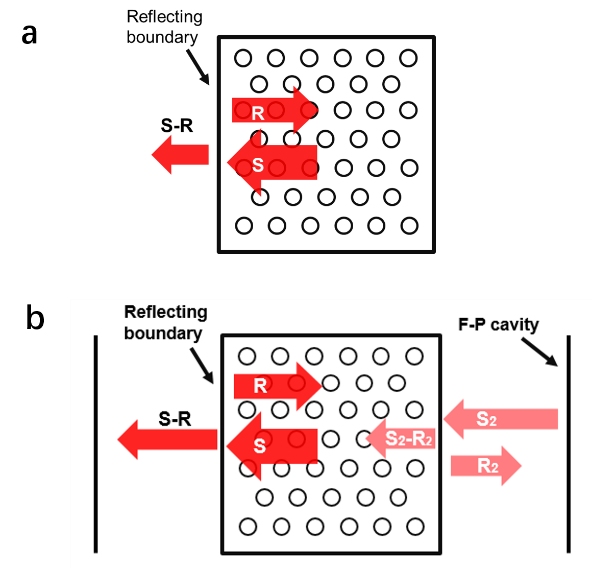


**Supplementary Figure S4. Schematic diagram of top-view of the PCSEL.** a, PCSELs without an FP cavity suffer loss of light at the edge of the photonic crystal. b, PCSEL with FP cavity. High reflectivity can be attained by the cleaved sides of the FP cavity. A hybrid cavity mode is formed by the FP cavity and photonic crystal.

In order to further increase the surface emission power of the PCSEL, we placed a mirror at one end of the waveguide, as shown in Figure S5. In addition, a cover was placed close to the mirror to block any stray light from the reflector being detected by the power meter. In Figure S6, we show the ratio of the output power of the surface emitting with the mirror and the one without mirror as a function of current. As can be seen that, after the threshold, the measured output power of PCSEL with mirror is around 1.7 times of the one without mirror. The reason is that the laser which emits from the cleaved sides will reflect to the waveguide by the mirror and then is refracted by the PC. Therefore, it is indicated that the higher output power of surface emitting can be realized by increasing the feedback of the light travelling in the waveguide. Our next step is to fabricate a nanostructure on the surface of the waveguide or coat HR films on both sides of the cleaved facets, to enlarge the feedback and achieve higher output power.


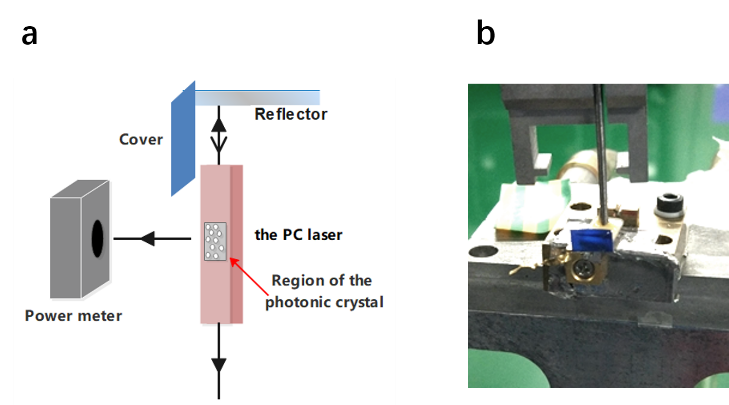


**Supplementary Figure S5.** **The measurement system for the PCSEL with mirror.** a, Schematic diagram of system. b, Photograph of the actual system.


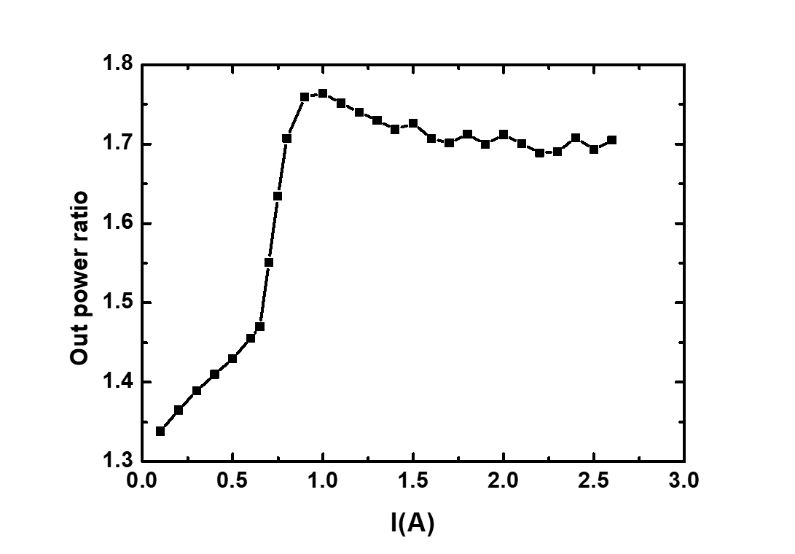


**Supplementary Figure S6. The effect of the external mirror.** The ratio of the output power of the PCSEL with and without the mirror as a function of current.

**5. Calculation of the characteristic temperature**

The threshold current density *J*_th_(T) at a given temperature T can be calculated by using the following equation [5-7]

$J_{\mathrm{th}}\left( T \right)=J_{\mathrm{th}}\left( T_{1} \right)\exp\frac{T-T_{1}}{T_{0}}$, (S1)

where *J*_th_(T_1_) is the threshold current density at temperature of T_1_. In our experiments, T_1_ is 295.15K, and T_0_ represents the characteristic temperature.

**6. Measurement of the far field of the PCSEL**

In Gaussian optics, transmission from point (r_1_,θ_1_) to point (r_2_,θ_2_) of a near-axisymmetric optical system can be calculated by the ray transfer matrix,

$\left( \begin{aligned} r_{2} \\ \theta_{2} \end{aligned} \right)=T\left( \begin{aligned} r_{1} \\ \theta_{1} \end{aligned} \right)=\left( \begin{matrix} A & B \\ C & D \end{matrix} \right)\left( \begin{aligned} r_{1} \\ \theta_{1} \end{aligned} \right)$. (S2)

When light passes through a thin lens with a focal length of f, A, B, C and D are 1, 0, -1/f and 1, respectively. The system for measurement of the far field intensity distribution of the PCSEL is shown in Figure S7. The laser spot on the plane of the CCD can be magnified or demagnified by adjusting the focal length and position of the lens F_2_. Therefore, we can obtain an image of the laser output, and the real size of the image on the focal plane in the x- and y-axes is 2.367 and 2.389 mm, respectively, corresponding to far field divergence angles of 16.8° and 17.0° in the horizontal and vertical directions, respectively. It should be noted that the larger FWHM is due to the oblique direction of the diffracted light in the vicinity of flat band.


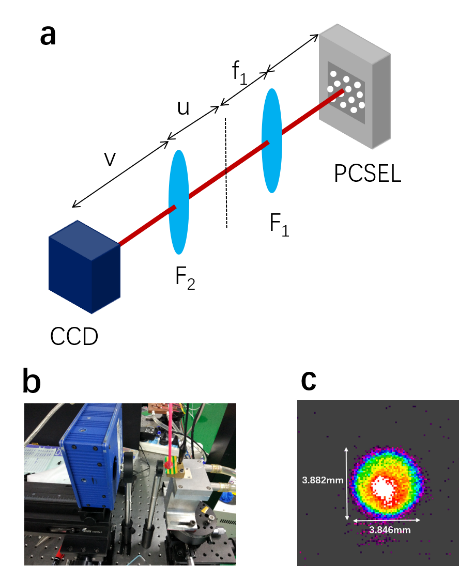


**Supplementary Figure S7.** **The system for measurement of the far field of the PCSEL and the obtained far field.** a, Schematic diagram of optical system. b, Photograph of the actual system. c, The far field of the PCSEL.

**References**

[1] Imada, M., Chutinan, A., Noda, S. & Mochizuki, M. Multidirectionally distributed feedback photonic crystal lasers. *Physical Review B* **65**, doi:10.1103/PhysRevB.65.195306 (2002).

[2] Gelleta J., Liang Y., Kitagawa H. and Noda S., “Influence of external reflection on the TE mode of photonic crystal surface-emitting lasers”, *Journal of the Optical Society of America B* **7**, 1435 doi: 10.1364/JOSAB.32.001435 (2015).

[3] Baumannm K., Stöferle T., Moll N., Mahrt R. F., Wahlbrink T., Bolten J., Mollenhauer T., Moormann C. and Scherf U., “Organic mixed-order photonic crystal lasers with ultras-mall footprint,” *Appl. Phys. Lett.* **91**, 171108, doi: 10.1063/1.2801706 (2007).

[4] Watanabe, A., Sugiyama, T., Kurosaka, Y., Hirose, K. and Noda, S. in 2013 Conference on Lasers and Electro-Optics Pacific Rim. ThI1_3 (Optical Society of America).

[5] Coldren, L.A. and Corzine, S.W. (Writers), Shi, H.X. (Translator) (2006) Diode Laser and Integrated Optics. Beijing University of Posts and Telecommunications Press, Beijing.

[6] X. Gao, Y. Qu, B. X. Bo, B. S. Zhang, X. D. Zhang, 808 nm High Power Semiconductor Lasers with High Characteristic Temperature. Semiconductor Optoelectronics, 6, 388-389 (1999).

[7] Lu Z. F., Wang L. J., Zhang Y., Shu S. L., Tian S. C., Tong C. Z., Hou G. Y., Chai X. L., Xu Y. Q. and Ni H. Q., “High-power GaSb-based microstripe broad-area lasers”, *Appl. Phys. Express* **11**, 032702 10.7567/APEX.11.032702 (2018).
